# The Double ‘V’ sign of endomyocardial fibrosis

**DOI:** 10.31744/einstein_journal/2026AI0595

**Published:** 2026-01-02

**Authors:** Kevin Rafael De Paula Morales, André Vaz, Rafaela Vieira Franklin, Gabriela Ribeiro Prata Leite Barros, Luiz Raphael Pereira Donoso Scoppetta, Eduardo Kaiser Ururahy Nunes Fonseca

**Affiliations:** 1 Universidade de São Paulo Instituto do Coração Faculdade de Medicina São Paulo SP Brazil Instituto do Coração, Hospital das Clínicas, Faculdade de Medicina, Universidade de São Paulo, São Paulo, SP, Brazil.

A 65-year-old woman presented to the cardiology department with exertional dyspnea and palpitations. Physical examination revealed bilateral jugular vein distension and mild lower-extremity edema. Blood tests indicated high B-type natriuretic peptide levels (777ng/mL). Echocardiography showed myocardial hypertrophy of the apical segments of both ventricles with cavity obliteration and left ventricular dysfunction with diffuse hypokinesia. Cardiovascular magnetic resonance (CMR) was performed to confirm the diagnosis. Cine images revealed biatrial enlargement, biventricular systolic dysfunction, and obliteration of both ventricular apical regions (Figures 1A and 1B, Video 1: https://youtu.be/ClHAKHkS2ek). Myocardial tissue characterization with late gadolinium enhancement (LGE) showed subendocardial enhancement of both ventricular apices (arrows in Figures 1C and 1D) and overlying thrombus (symbol (*) in Figures 1C and 1D) of the left ventricle (double "V" sign). Myocardial strain evaluated using the feature-tracking technique revealed an apicobasal gradient with reduced mobility in the apical segments. Although some basal segments did not show completely normal strain compared to the reference values, myocardial strain was significantly more impaired in the apical region. This highlighted the described gradient with greater impairment in the apical region.^(1)^ (Figure 2, Video 2: https://youtu.be/XOnc7CF18K0). Cardiovascular magnetic resonance findings were consistent with those of endomyocardial fibrosis (EMF).

**Figure 1:**
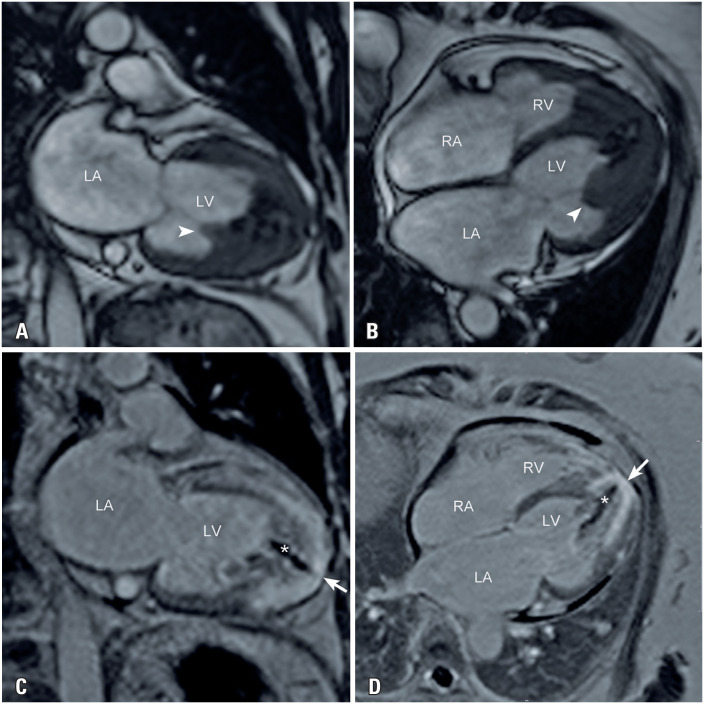
Steady-state free precession cine images in the (A) two-chamber and (B) four-chamber views, and inversion-recovery late gadolinium enhancement images in the (C) two-chamber and (D) four-chamber views

* Thrombus.

Arrowhead: Posteromedial papillary muscle, Arrow: Subendocardial LGE.

RA: right atrium, LA: left atrium, RV: right ventricle, LV: left ventricle.

**Figure 2:**
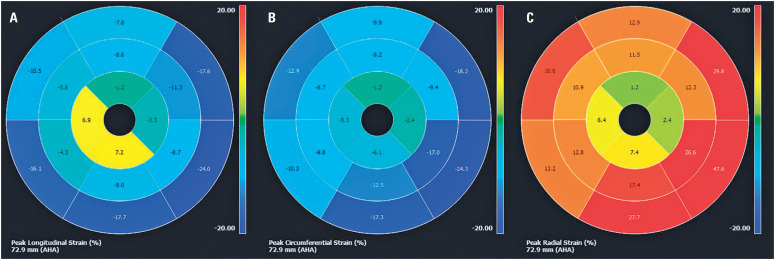
Feature-tracking myocardial strain demonstrates an apicobasal gradient with reduced mobility of the apical segments. The peak longitudinal strain (A), peak circumferential strain (B), and peak radial strain (C) were −5.3%, −9.6%, and 13%, respectively

Endomyocardial fibrosis is a major cause of restrictive cardiomyopathy in tropical regions, particularly in underdeveloped countries.^(2)^ Although the exact etiology is not well known, the disease is characterized by fibrotic tissue deposition in the endocardium of one or both ventricular apices.^(2)^

Cardiovascular magnetic resonance imaging provides detailed information on the ventricular morphology and excellent visualization of the ventricular apex. LGE can precisely evaluate myocardial fibrosis with typical patterns of distribution, leading to a more accurate diagnosis.^(3)^ A study suggested that CRM provides prognostic information.^(3)^

Common CMR findings include small ventricular cavities and apical obliteration, which are frequently associated with thrombus formation and calcification.^(2)^ Extension to the inflow tract and involvement of the atrioventricular leaflets are common.^(2)^ The most typical CMR finding is the double "V" sign seen in the LGE sequences, which consists of subendocardial enhancement and an overlying thrombus at the apex.^(2)^

This case demonstrates that CMR is a valuable tool for the evaluation of EMF and is useful for differential diagnosis of left ventricular apical hypertrophy and obliteration. Incorporating myocardial strain analysis enhances our understanding of cardiac function by enabling the early detection of regional abnormalities and aiding in the differentiation of conditions, such as EMF, from other apical cardiomyopathies.

## Data Availability

The underlying content is contained within the manuscript.
